# Life as an early career researcher: interview with Eleftheria Anastasopoulou

**DOI:** 10.4155/fsoa-2016-0013

**Published:** 2016-03-03

**Authors:** Eleftheria A Anastasopoulou

**Affiliations:** 1 Cancer Immunology & Immunotherapy Center (CIIC), Saint Savas Cancer Hospital, Athens, Greece

**Keywords:** antigen-specific T cells, biomarkers, cancer vaccines, HER-2/neu, multiparameter flow cytometry

## Abstract

**Eleftheria Anastasopoulou speaks to Francesca Lake, Managing Editor:** Eleftheria is currently pursuing her PhD in immunotherapy of cancer and biomarkers. Her interests focus mainly on detection of tumor antogen-specific T cells in vaccinated patients. She attained her degree at the National and Kapodistrian University of Athens, and holds an MSc from Leiden University.

## Q Can you tell us about your career path to date?

I started my studies in the Biology department in the University of Athens (Greece). During that time I worked as an intern in the Biochemistry department at the General hospital ‘Evaggelismos’, where I became familiar with the daily routine in a hospital. I performed my research thesis in the field of molecular neurobiology and Alzheimer disease in transgenic mouse models, at the Biomedical Research Foundation of the Academy of Athens (BRFAA), where I also worked as a lab technician after my graduation. Having a major degree in Biology, I decided to pursue a Masters in Biomedical Sciences with management specialization in Leiden University, in The Netherlands. During my postgraduate studies, I had the opportunity to perform my main research project at Leiden University Medical Centre (LUMC) in the field of immunology, investigating virus-specific immune responses (CMV, EBV, among others). My project leader introduced me to the science of immunology and through this internship I realized I wanted to continue to work in this research field. Thus, I decided to participate in a student exchange program in Heidelberg, where I followed specific training on tumor immunology, virology and cancer at German Cancer Research Center (DKFZ). During that period, I had the opportunity to work at the Department of Epigenetics and Cancer Risk Factors at DKFZ, where I became familiar with novel experimental techniques in cancer epigenetics. Having successfully completed my exchange program, I did my management internship in the field of clinical trials at Centre for Human Drug Research (CHDR) in Leiden Bioscience Park. Working as a junior project leader, I learned basic aspects of clinical trial design and GCP rules and, most importantly, how to collaborate with people of different disciplines. After receiving my Master's, I decided to return to Greece where I started my PhD project on cancer immunotherapy and biomarkers, at the Cancer Immunology and Immunotherapy Center (CIIC) in Athens. CIIC is situated in the St Savvas Cancer Hospital and it is the only institute in Greece working in the field of immunotherapy.

During the last few years, I have enriched my background in tumor immunology and upgraded my technical skills in flow cytometry. Through my research, I realized that applying flow cytometry in the field of immunology is something that makes me thrilled and fascinates me. So, I constantly try to improve my skills through participations in seminars and proficiency panels.

## Q What are you working on at the moment?

Currently I am pursuing my PhD in Cancer Immunotherapy. I am trying to identify biomarkers of prognostic and/or predictive significance for patients that could benefit from immunotherapy with a HER2/neu peptide vaccine. Considering that several cancer vaccination trials have failed due to the clinical design, identifying biomarkers for patient selection is of utmost importance. Additionally, I am investigating specific cellular immune responses in the context of vaccine evaluation. I am using multiparameter flow cytometry in combination with multimer staining, to identify vaccine-induced T cells and further characterize them based on their memory status. Although multiparameter flow cytometry can be really tricky and challenging, it is really fascinating how it unveils the complex phenotype of memory subsets and antigen-specific subpopulations.

## Q What are your career goals over the next 20 years?

This is a difficult question, especially when it comes to the research field. First of all, I am planning to finish my PhD. Then, I would like to continue my research in tumor immunology. However, there are several factors that sometimes limit your options. For instance, I would like to continue my research in an accredited institute specialized on cancer research, such as the German Cancer Center (DKFZ) in Heidelberg, or The Netherlands Cancer Institute (NKI) in Amsterdam, but unfortunately in Greece there is not that kind of institute. Nothing would make me happier than to be able to work in my country and offer my knowledge in the field on tumor immunology.

## Q What steps do you expect to take to get them?

Considering the difficult financial situation in Greece, it is not easy for young researchers to find their own funding for their research projects, but I assume this is also a common issue in other countries too. Hence, I would like to apply for a research grant from European or international sources, so as to continue my research career in my country. However, given the situation, I cannot exclude the possibility of pursuing a job opportunity abroad.

## Q What would you say are the biggest challenges facing you as an early career researchers?

One of the biggest challenges as an early career researcher is the ability to implement your research ideas. This includes funding support but also getting the right colleagues. It is not easy to collaborate with people that do not share a team spirit or are very competitive. What I have learned, through my internships in several working environments, is that team spirit is the most important factor for ensuring good collaboration.

## Q How would you suggest these are tackled?

As far as the funding support is concerned I guess a young researcher should be patient and optimistic. Usually, good ideas are rewarded. Regarding the collaboration, I believe that laboratory rotations and internships are necessary. First of all, they can assist a young researcher in making a decision about what he/she wants to do in his/her research career and build a scientific network. Second, working with different people in different laboratories is really advantageous for a young scientist, since it favors the development of the team spirit and provides the necessary assets for adapting in new working environments.

## Q If you could go back 5 years, what advice would you give to your younger self in terms of your career?

I would say to myself to: ‘trust your instinct’. 5 years ago, I was working as a laboratory technician in a Cancer Immunology and Immunotherapy center. I was working on a research field I like and I was satisfied with my colleagues, so when I was offered a PhD position, the answer was easy, ‘yes’. I believe that the lab environment is very demanding and really stressful sometimes, so people often show their ‘bad’ character. Hence, before starting a PhD or even an internship, it is important to spend a few weeks in a lab and see if the people and the environment actually ‘suit’ you. I always bring to my mind the words of an old colleague, when we were attending a course in Utrecht University: “It is not the project that really matters but the people you have to deal with every day for the next 4, 5 or even more years of your life”.

